# Transparency as design publicity: explaining and justifying inscrutable algorithms

**DOI:** 10.1007/s10676-020-09564-w

**Published:** 2020-10-20

**Authors:** Michele Loi, Andrea Ferrario, Eleonora Viganò

**Affiliations:** 1 Institute of Biomedical Ethics and History of Medicine, University of Zurich, Zurich, Switzerland; 2 ETH Zurich, Zurich, Switzerland

**Keywords:** Machine learning, Transparency, Explanations, Justifications, Philosophy of science, Computing methodologies ~ Artificial intelligence, Cognitive science, Machine learning, Human-centered computing ~ HCI theory, Concepts and models

## Abstract

In this paper we argue that transparency of machine learning algorithms, just as explanation, can be defined at different levels of abstraction. We criticize recent attempts to identify the explanation of black box algorithms with making their decisions (post-hoc) interpretable, focusing our discussion on counterfactual explanations. These approaches to explanation simplify the real nature of the black boxes and risk misleading the public about the normative features of a model. We propose a new form of algorithmic transparency, that consists in explaining algorithms as an intentional product, that serves a particular goal, or multiple goals (Daniel Dennet’s design stance) in a given domain of applicability, and that provides a measure of the extent to which such a goal is achieved, and evidence about the way that measure has been reached. We call such idea of algorithmic transparency “design publicity.” We argue that design publicity can be more easily linked with the justification of the use and of the design of the algorithm, and of each individual decision following from it. In comparison to post-hoc explanations of individual algorithmic decisions, design publicity meets a different demand (the demand for impersonal justification) of the explainee. Finally, we argue that when models that pursue justifiable goals (which may include fairness as avoidance of bias towards specific groups) to a justifiable degree are used consistently, the resulting decisions are all justified even if some of them are (unavoidably) based on incorrect predictions. For this argument, we rely on John Rawls’s idea of procedural justice applied to algorithms conceived as institutions.

## Introduction

In this paper, we provide a new theory of algorithmic transparency, with a focus on both explanations and justifications, where we consider as “algorithms” those human artifacts stemming from the training of machine learning models on digital data, in order to generate predictions to assist or automate decision-making. These algorithms are subject to intense scrutiny for both technical and moral reason, as their applications in product and services is constantly increasing, as well as their potential to affect everyone’s lives. Examples come from credit scoring, to digital financial coaching and job assistants, automated insurance claim processing bots, smart home services, online dating platforms, autonomous driving solutions and policing as well as recidivism scoring algorithms. One current limitation of modern algorithmic-assisted decision-making is that most advanced machine learning models are considered as “black boxes” or inscrutable (Selbst & Barocas 2018). Therefore, the last few years have seen the rise of an active debate in the scientific community around interpretability, transparency, explainability and justification of (machine learning model-based) algorithms and their outputs. Without a proper understanding of these constructs and their outcomes, any decision generated or supported by these algorithms cannot be adequately contested.

According to (Lipton 2018), interpretations of machine learning models fall into two categories: model transparency and post-hoc explanations. Model transparency is “some sense of understanding the mechanism by which the model works”(Lipton 2018). We claim that transparency (at least, the kind of transparency we characterize in this paper) is valuable because and in so far as it enables the individuals, who are subjected to algorithmic decision-making, to assess whether these decisions are morally and politically *justifiable*. We explain the relation between transparency and justification in “Design Publicity and Justification.” Different ideas may be conveyed by demanding that a machine learning model be transparent, each focusing on different aspects of the model, its components and the training algorithm (Lipton 2018). On the other hand, post-hoc explanations focus on the outcome of the (learned) model; they include (Mittelstadt et al. 2019) natural language processing explanations, visualizations, case-based and counterfactual explanations, and local approximations; they can be classified in model specific or model agnostic. Local approximation allow, in particular, to explain why a black box model produced a selected prediction by approximating it with an interpretable model (e.g. a linear regression) around the prediction at hand (Ribeiro et al. 2016). We refer to the goal of post-hoc explanations of individual decisions as “model interpretability.”

In this paper, we advance a new conceptualization of what explaining an algorithm amounts to. A key feature of our proposal is that we are fully explicit about the purposes that this mode of explanation is intended to achieve. Design publicity is intended to empower the public to debate all the key algorithmic design and testing choices relevant to assessing whether the decisions taken by such systems are justified. This enables the revision of such design choices as the public understanding of these ideas evolves. It is not intended to identify solutions deemed absolutely correct and incorrigible, to be enshrined once and forever in code. Justification will realistically always be imperfect and incomplete in spite of the best efforts put into it.

Design publicity takes the perspective of society (or of a regulator on behalf of society) rather than that of an actual, *concrete,* particular individual subject to algorithmic decisions. It does not ignore that society is made of individuals, but it assumes that individuals are able to take (or at least aspire to) an impersonal standpoint when judging such systems. We refer to the more abstract idea of impartiality, not to a specific account of it—on which contrasting philosophical proposals have been put forth—to characterize the type of perspective that we have in mind here.^1^

Given this normative standpoint, we address a specific limitation of those post-hoc explanations that identify a feature or limited set of features as the reason(s) why a decision about an individual case was made. Although the post-hoc mode of explanation can be useful to empower individuals in certain settings, we argue that it fails to explain why a decision based on certain features is normatively adequate and justifiable from an impartial standpoint. While post-hoc explanations do not provide useful elements to judge and debate the justification of such systems, we intend transparency as design publicity to enable that type of discussion.

In this paper, after providing some definitions (2), we highlight some limitations of interpretable algorithms, by giving a prominent example of post-hoc interpretability methods, i.e. counterfactual explanations, drawing support from the recent literature. We, then, (3) propose a new concept of algorithmic transparency that overcomes the classical split in model transparency vs. interpretability and which we label “transparency as design publicity”; subsequently, (4) we argue that it provides a kind of explanation of their behavior: a teleological explanation, or explanation by design. This form of explanation tries to take into account the domain-specificities of the algorithm as well as the expertise, understanding and interests of its end-users. The special value of this explanation is that it links the behavior of an algorithm to their justification (5) and, when the algorithm is used consistently, to the procedural justice of its decisions.

### Machine learning and algorithms: some definitions

In this section, we introduce some definitions that are relevant for the remainder of this contribution. The aim here is to provide the reader with an overview of some commonly used concepts in the most recent literature on philosophy of technology and artificial intelligence without indulging (too much) in technicalities and jargon. We start with *machine learning*, which is a multidisciplinary discipline “concerned with the question of how to construct computer programs that automatically improve with experience” (Mitchell 1997). Machine learning draws on concepts from artificial intelligence, information theory, algorithmics and philosophy, among others. A machine learning problem “can be precisely defined as the problem of improving some measure of performance P when executing some task T, through some type of training experience E” (Mitchell 1997). Training experience E is represented by (digital) input data, which are preprocessed and formatted for the machine learning problem under consideration. Performance measures P can be off-the-shelf or ad-hoc, that is engineered by the resources responsible for the solution of the corresponding machine learning problem; they provide with an estimation of the error made by the solution to the machine learning problem in executing T, using experience E.

Solving a machine learning problem consists of specifying a class H of mathematical constructs called *machine learning models*, to be trained on input data D using a set of *algorithms* implemented in computer-understandable programming languages (Mitchell 1997). Therefore, through the algorithms in the training process, the best machine learning model is trained or learned. The result of this process is an *object* in a programming language embedded in an IT infrastructure to generate predictions on new data with the goal to assist or automate decision-making; such complex, dynamic computer system becomes a “cognitive engine” at the core of products and services mentioned in (1). In the remainder of these notes, we will call this object “algorithm;” in fact, this is an algorithm—i.e. a procedure or rule to compute predictions from input (new) data points—and stemming from the training of machine learning models to solve a given machine learning problem. We will come back to the teleological nature of algorithms in (4).

### Post-hoc explanations of machine learning models

As discussed by Selbst and Barocas “interpretability has received considerable attention in research and practice due to the widely held belief that there is a tension between how well a model will perform and how well humans will be able to interpret it” (Selbst & Barocas 2018). Following Lipton (2018), we refer to post-hoc interpretability as the provision of understandable explanations of machine learning model outcomes, also called predictions. Despite the proliferation of post-hoc interpretability tools in the literature of explainable artificial intelligence, we now explain more theoretically, with reference to prior work (Selbst & Barocas 2018), what post-hoc interpretability explanations intend to achieve and why they lead to partial understanding of the impersonally salient normative features of algorithmic systems.

We shall focus on a prominent example of explanations for post-hoc interpretability of machine learning models, i.e. counterfactual explanations, which recently drew attention in the artificial intelligence research community (Wachter et al. 2017). But we focus on limits to this approach that are shared with other post-hoc explanation.^2^ Counterfactual explanations are (1) “a novel type of explanation of automated decisions that overcomes many challenges facing current work on algorithmic interpretability and accountability” (Wachter et al. 2017), (2) “should be used as a means to provide explanations for individual decisions” (Wachter et al. 2017), and (3) “can bridge the gap between the interests of data subjects and data controllers that otherwise acts as a barrier to a legally binding right to explanation” (Wachter et al. 2017). For simplicity, we do not consider the theory of counterfactuals and causality, limiting our considerations to machine learning counterfactuals only.

Counterfactual explanations identify the explanation of machine learning outcome by the provision of a set of factors, or model features, whose change in value alters the outcome under consideration, keeping all other factors equal (Wachter et al. 2017). By design, they highlight “a limited set of features that are most deserving of a decision subject’s attention” (Barocas et al. 2020). Therefore, in the best-case scenario, counterfactual explanations provide users with actionable strategies to change the outcome into a more favorable one (recourse) as a response to a machine-generated decision (Ustun et al. 2019).^3^

For the purpose of our discussion, the most salient limitation of counterfactual explanations, shared with other post-hoc explanations, follows from their being “feature-highlighting” (Barocas et al. 2020), i.e. these explanation provide “an explanation that seeks to educate the decision subject by pointing to specific features in the model that matter to the individual decision” (Barocas et al. 2020, p. 81).^4^ This way of educating the decision subject is silent about the reasons why the model makes the decision based on such (and other) features, in the first place, e.g. why individuals in general are judged by such features. But this is highly relevant for one to evaluate whether the decision based on such features is justifiable. The explainee of counterfactual explanations must accept as a presupposition that the decision is (reasonably?) taken based on certain features. The explainee is modelled as having exclusively self-centered, concrete, pragmatic interest in the specific features that are relevant for the decision about her or him. So the explainee is not provided with essential elements necessary to judge whether taking such decisions based on certain features is socially desirable or acceptable. In this context, the justification of the algorithmic practice is treated as irrelevant to the explainee.

Counterfactual explanations lack normative informativeness, as it is not possible to infer normative properties of the model from explanations of individual decisions. As will be later argued, the most normatively important properties of model-based decisions emerge from repeated application of the model—they are properties of the kind of patterns, e.g. the distribution of errors, or of benefits, between groups, or of groups defined by morally salient properties, that emerge when the law of large numbers applies, as well as the model accuracy and ability to generate benefits (e.g. profit, or other forms of utility). A case in point of a morally salient property is indirect discrimination or disparate impact, which can be considered morally or legally relevant in certain contexts, but cannot be determined by reference to counterfactual explanations, because a protected group may be discriminated via proxy (e.g. ZIP code can be a proxy of race), so the protected group information will not appear as a feature in the model. Interestingly, the opposite misunderstanding may also occur. A counterfactual explanation may show that a decision, e.g. concerning a loan, would have been different had the individual been of a different race. This explanation may suggest unfairness, even when the intention behind using information about the protected group is used to make the prediction fairer, e.g. the information is used to ensure the statistical property of separation (Hardt et al. 2016).

Summing up, counterfactual explanations do represent a practical strategy to explanation in presence of few variables and scenario choices, where the assumption that the model makes decisions that are normatively appropriate is taken for granted. In what follows, we provide a model of transparency that relies on explanations that are relevant for the justification of algorithmic decisions and, thus, their public acceptability.^5^ We do not maintain that transparency as design publicity—the approach we propose—fulfill all the desiderata various authors have associated with explainable and interpretable AI. Our transparency idea serves a particular purpose: that of normative justification. It provides the kind of explanation, which is useful for the public to assess if the deployment of algorithmic decision-making and the decisions following from it is justifiable.

### Design explanation of algorithms

As showed by Kroll (2018), the thesis that the understanding and transparency of algorithmic-assisted decision-making is limited by the inscrutability of the machine learning models and their algorithms (i.e. the fact that they are opaque or “black boxes”) is criticizable. The debate on algorithm inscrutability mostly depends on the meaning we attribute to the expression “explaining the model” and accordingly “understanding the model.”

Explanation—the process and product (Ruben 2012) of making something understandable—has many meanings: definition, interpretation, individuation of the necessary conditions or sufficient conditions, of purposes, of functions, and of goals. An explanation is effective when the *x* that is explained is clear and open to people that want to understand *x*. An effective explanation renders an object understandable and its understandability contributes to the transparency of the object, i.e. the quality of being easy to see through, analyze, and assess.

The explanation of the behavior of an algorithmic system has not only different meanings but also different levels of abstraction to which it can refer (Floridi & Sanders 2004). For example, if we consider a low level of abstraction for the algorithmic system by focusing on its core mechanics, then explainability strategies will focus on its functioning, both from a theoretical (e.g. considering the machine learning model, including the optimization procedures for learning) and more engineering-oriented (e.g. the software running the machine learning training and the algorithm deployment) perspectives. However, at this level of abstraction, explainability may be difficult to reach even to computer scientists and engineers (Lipton 2018). On the other hand, one could consider a level of abstraction where explanations clarify *the purpose* of algorithms; these would be understandable to the public, from the end users with low expertise to policymakers in the need of justifying the use of algorithmic-assisted decision-making, to corporate executives adopting such models, to computer scientists and engineers that design them.

We define a design explanation of an algorithmic system to be the explanation of what such a system does, which essentially describes the ability of a system to achieve a given purpose. The design explanation of an algorithmic system is an explanation by intelligent design, namely it explains an *x* by referring to that for the sake of which *x* was created. This explanation is more abstract than the mechanistic one and corresponds to Dennett’s design stance, namely the intellectual strategy by which we explain the behavior of a system by referring to its purpose and intentional design (Dennett 1987). Design explanations are teleological and focus on the final cause of a system (Aristotle 2016, 2018).^6^ Design explanation is applicable to algorithms as the latter are goal-directed, human artifacts produced in a specific sociotechnical context (Baker 2004). In the design explanation of a common object such as a chair, we provide the reasons for which the chair was designed as such: being stable and comfortable; these goals directed the design of the chair and explain why respectively the chair has four or three legs and has an ergonomic or flat surface in the spot in which we sit down. The design explanation of an algorithm comprises “the understanding of what the algorithm was designed to do, how it was designed to do that, and why it was designed in that particular way instead of some other way” (Kroll 2018). In other words, explaining the purpose of an algorithm requires giving information on various elements: the goal that the algorithm pursues, the mathematical constructs into which the goal is translated in order to be implemented in the algorithm, and the tests and the data with which the performance of the algorithm was verified.

We define the *design transparency* of an algorithmic system to be the adequate communication of the essential information necessary to provide a satisfactory design explanation of such a system.^7^ As design explanation is made of different elements, so design transparency can be split into various components: *value transparency*, *translation transparency*, and *performance transparency*, as we will now show.

The goal of an algorithm is something valuable that is achieved. Since it is something that is desired by a person or group, we can also call it a good or *value* for that person or group. Value transparency should also indicate why and for whom the goal is valuable, when this is not obvious from the context. The design *goal* (e.g. identifying the most profitable clients, minimizing hospital readmissions) is typically also the *goal* of the person who decides to employ the artifact in practice. Thus, it also figures in the *intentional explanation* of the action to develop, or purchase, and employ the AI, by the persons accountable for such decisions. Thus, the design explanation should indicate which is the goal—the reasons or motivations—of the computer scientists and engineers who designed the algorithm and of the persons accountable for its employment in real-world settings. These goals should be one and the same; when this is not the case, the artifact does not respond to the reasons of the person who are supposed to have meaningful human control (Santoni de Sio & Van den Hoven 2018) over it. This is problematic for accountability. The goal of an algorithm is usually a practical objective, such as profit or efficient allocation of scarce resources, but can include moral values such as equity, beneficence, trustworthiness, and the rules that are socially accepted as pertinent for the domain in which the model is employed. In both cases, the goal introduces normativity in the model, as it represents something that there are good reasons to pursue. Hence, normative choices are made both when normative standards are explicitly invoked in the design of a model and when they are ignored. As Binns points out:

[W]hen attempting to modify a model to remove algorithmic discrimination on the basis of race, gender, religion or other protected attributes, the data scientist will inevitably have to embed, in a mathematically explicit way, a set of moral and political constraints [6].

The goals or values that guide the design of algorithmic models should therefore be included in an explanation of such models. *Value transparency* is the result of an explanation that makes the standards, norms, and goal that were implemented in the system accessible. These normative elements should also correspond to the *reasons for which* it was deployed.

The goal of an algorithmic system needs to be translated into something that is measured: a set of rules with which the algorithm elaborates inputs and produce outputs. A machine learning algorithm requires the quantification of the goal because, in particular, the algorithm that generates the model needs to quantify the departure from the model objectives of several potential candidate models. There is no straightforward and unique way to translate a goal into a mathematic construct. For this reason, making such translation a publicly verifiable criteria provides the public and scientific community with the information to assess how a given goal is operationalized in machine-language. Making this piece of information public constitutes *translation transparency*, which is part of design transparency. In applications, it is possible to have alternative translations in machine language of the same goal. For example, let us consider the problem of designing a predictive model of customer churn^8^ for an airline company. The goal is to design and implement a predictive model of customer churn in order to assess future profitability of a given portfolio of customers. However, in the case of an airline company, the business concept of “churn” could be translated into a different set of computer-understandable rules. In one case, one could simply define a customer as churned if no revenue is generated by the customer in a given year of interest. On the other hand, one could introduce churn as the absence of revenue in a given year of interest and the lack of flying activities (i.e. avoiding the case of zero-revenue generated by customers flying using promotions). Both choices lead to alternative implementations of the same business goal. Design transparency recommends to explain the definition of churn and its motivations to the public. Another example is the goal of fairness or avoiding discrimination. Different definitions of fairness for predictive models exist and it is often impossible to satisfy all of them simultaneously (Berk et al. 2018). Design transparency requires declaring which fairness definition has been adopted, and, if possible, to provide a justification of such choice.

Once the criteria to measure the degree of goal achievement are specified, a design explanation of an algorithm should provide information on the effective achievement of such objectives in the environment for which the system was built. In fact, for instance, the mere implementation of the most advanced norms of equal treatment in a credit-granting system does not guarantee that the system will be effectively impartial. The impact of the algorithms and its outcomes needs to be considered. *Performance transparency* consists in indicating the logic with which the algorithm has been tested in order to verify how much it departed from achieving the goal and in indicating the results of such logic, starting with the choice of performance measures used in both training phase and during the assessment of the model on test data.^9^ These latter are data that have not been used during training and whose scope is to assess the adaptability of the model to unseen inputs. The test data are part of performance transparency as the choice and the quality of them, which can be subject to biases, influences the performance measure and thus the assessment of the algorithm. If performance assessment is not robust in contexts with a different causal regime (e.g. hospital data from Brasil vs. from Japan), transparency about the test data may reveal limitations of performance claims.

In summary, an algorithmic system has the property of design transparency if and only if it provides the public with the goal of the algorithm (value transparency), how this goal was translated into programming language (translation transparency), how the algorithm rule achieves that goal and how the goal achievement has been assessed (performance transparency). The division of design transparency into three components (value, translation, performance) enables the analysis of the algorithm at three different levels of explanations, which, as we will see, constitute also three levels that a design explanation has to address.^10^

We illustrate with an example what the approach of design transparency requires in practice. We shall argue only in the next section that such design transparency is essential for design publicity, i.e. for debating the justifiability of the algorithmic practice in question. The owners of a stadium have to decide whether to adopt a face recognition (FR) system at the entrance of the stadium to prevent terroristic attacks.

For *value transparency,* the FR decision to block an individual is explained by pointing out the design goals of the system. The primary goal is to prevent a terrorist attack in a place in which many people gather. Moreover, it is likely that the goal of the system includes the properties that the system is reliable, fair, and avoids excessive disruptions to the use of the stadium.

For *translation transparency*, the owner of the stadium, as well as independent auditors and legal authorities, should have easy access to a lower-level (i.e. more detailed) description of the implementation of the goal and constraints in the FR system. Thus, the vendors of the FR systems should make public that the system detects faces in real time and compares the faces of people at the stadium entrance with others stored in a database containing the pictures of terrorists, by extracting facial features. The vendor should also explain the mathematical balance between the goals of security and non-disruption of business, e.g. that the social disutility of allowing an actual terrorist in (a false negative) is regarded as equivalent to that of preventing an actual non-terrorist from seeing the match (a false positive). Thus—the vendor could explain—the algorithm is trained to maximize classification accuracy (i.e. the percentage of correct predictions), ignoring the distinction between type-I and type-II errors.^11^ Furthermore, let us assume that the fairness of the system is translated with the mathematical notion of equality in the false-positive and false-negative rate across all the legally defined race and gender groups.

For *performance transparency* the vendor should provide actual measures of the relevant performance metrics, that should be coherent with the translation assumptions above (i.e., classification accuracy and a meaningful comparison of group-related false-positive and false-negative rates). To make sense of the robustness of performance measures, the vendor should also provide information about the type of data with which the system was trained, whether the data were tested for possible biases (e.g. if there were only few faces of a given ethnicity), the confidence level of the human coders classifying the pictures with which the system was trained, and the contexts in which the system works better (e.g. with pictures of man instead of woman, with good lighting and high resolution only). Additionally, to achieve performance transparency, one would require information on how the system performance was assessed (e.g. disclosing information on the partition of data into training and test subsets), including the specification of whether the performance was assessed in the same context used for training or in a totally different environment. Notice that in this example, every level of design transparency consists of objectionable claims, exposing accountable parties to criticism by the experts and the broader public. E.g. security experts may object that false negatives are far more important than false positives, ethics experts may object to translating fairness, in this context, as equality in the false positive and false negative rate, and NGOs may point to racial biases in the way databases of terrorist faces are built.

An important step in addition to design transparency concerns the explanation of the singular decisions by the artifact, which should be distinguished logically from the nature of the artifact itself. The algorithm’s performance connects the explanation of the artifact (i.e. an algorithm, or rule) with the application of the rule to particular cases. The simple solution is to view each individual decision as a means through which the artifact achieves the overall goal for which it has been designed. This explanation is however problematic in the light of the fact that, when algorithmic decisions are based on statistical predictions, they will often fail to decide in a way that directly promotes the goal the model is designed to achieve. E.g. a loan is refused to someone willing and able to repay it, an inmate who will not reoffend is denied parole, a patient is prematurely released from the hospital, causing readmission. This is because decisions based on imperfect predictions about stochastic events will typically be often wrong, but sufficiently often right to justify the use of the model in practice. In the next section we are going to show why even the statistical imperfection of a model can be justified by appealing to its design goals and the trade-offs between all values pertaining to the justification of its use.

There is a further type of transparency—*consistency transparency—*that contributes to explain individual decisions by algorithms, given the assumption that the employment of such systems should be minimally fair. Consistency transparency is showing proof that consistency is achieved, i.e. that the algorithm always generates predictions by the same rules even when we cannot observe those rules in operation. Consistency is not a feature of the model but of its *deployment*. It does not contribute to explain why the model works in a certain way, but why certain decisions are made (namely, they result from applying the model consistently). Consistency can even be a property of the deployment of an algorithm that applies a discriminatory rule such as filtering job candidates by their residence address. Nonetheless, as consistency transparency shows that identical cases are treated identically, it represents the first step towards fairness; it is a sort of basic requirement of fairness that, as we shall show, is necessary but not sufficient to justify each decision as fair.

In some cases, models are *unidentifiable*, by which we mean that in most AI powered solutions the underlying machine learning models are updated (i.e. retrained) with frequencies that depend on the domain of applicability of the solution itself. This implies that an AI potentially generates different outcomes for the same end user, depending on the moment at which the outcome is generated: any explanation of this outcome (for the purpose of contesting or auditing it, for example) depends on time, as well. Consistency transparency requires that changes in a model be declared because, as we shall maintain, this is relevant for their justification. Consistency is a normative goal and showing that it is achieved by the model contributes to explaining why an individual decision is made–namely, by showing that it is explained by a normative consideration. Conversely, the failure to satisfy consistency implies that the decisions of the model can be challenged on a specific normative ground.

In conclusion, the design explanation of the model shows that an algorithmic model gives a decisional outcome because the model pursues a certain goal (value transparency), which is translated into mathematical constructs implemented in the algorithm (translation transparency), which in turn enables one to verify whether the model achieves the goal (performance transparency). When, as in most cases, consistency is among the reasonable goals of model *deployment*, the explanation of the *decisions* by the model includes consistency transparency. We hypothesize that in achieving design transparency, one can take into account domain-specific features of algorithms, as well as the level of expertise, knowledge and interests of their end-users. These factors are currently considered important in the scientific debate around explainable AI, model transparency and interpretability.^12^ This, together with the use of results from psychology and cognitive science to improve the understanding of the processes behind model interpretation by end-users (Miller 2019), represents a viable strategy to avoid what Miller et. al. refer to as “inmates running the asylum” (Miller et al. 2017).

### Design publicity and justification

We define design publicity as adequate communication of the essential elements needed for determining if a decision driven or made by an algorithmic system is justified. (The judgments in question are meant to be impartial and to enable an informed public discussion about the use of such systems.)

In what follows, we argue that both design transparency and consistency transparency, as defined in “Design explanation of algorithms,” are necessary for design publicity, because they are necessary to assess if and how the decision taken by (or with the help of) an algorithmic system is justified (when it is).

Design publicity provides information about (a) the goal the algorithm is designed to pursue and the moral constraints it is designed to respect (value transparency); (b) the way this goal is translated into a problem that can be solved by machine learning (translation transparency); (c) the performance of the algorithm in addressing problem (performance transparency); and (d) a proof of the fact that decisions are taken by consistently applying the same algorithm (consistency transparency). Let us now consider how each of these elements contributes to the justification of using an algorithm and of the decisions that follow from its use.

Let us begin with the goal or goals the algorithm is designed to pursue. All algorithms are designed to pursue a *primary goal* (e.g. a business objective); some more advanced algorithms are also designed to take into consideration a *plurality of different values*, such as fairness or privacy that often can be conceptualized as moral or legal *constraints*. *Constraints* are typically in trade-off with the primary goal and affect the way and the extent to which the primary goal can be achieved. For the sake of simplicity, we will refer to both goals and constraints (as goals), in what follows.

The first step of the *justification* of decisions taken by an algorithm, thus, requires evaluating the goals and constraints that the algorithm, respectively, achieves and respects. In a justified algorithm, they reflect those values and constraints a reasonable person may want to see promoted/respected in the context of a service.

The *primary goal* of the algorithm is essential to show that decisions are not morally arbitrary. Publicly recognized forms of social utility, such as security (in the FR example) or profit may fill this purpose. Primary goals matter to justification when they are *valuable* goals, e.g. there are good reasons to pursue such goals, which can be explained by reference to values commonly accepted in society, including moral, political or legal values, as well as the profit generated by free market exchanges, in capitalist societies that rely on profit as the motive stimulating socially efficient economic activity, resource allocation, and risk taking. Other goals (the “constraining” goals) typically reflect value considerations, e.g. privacy or fairness. Different types of justification are possible, for example in terms of common or philosophical morality, of the law, of by virtue of political principles and values that may be universal or characteristic of the society in which the model operates. Take, for example, anti-discrimination as the general name of a value that society expects from a FR service, and that contributes to define the goal of the algorithms (in this case, by constraining the distribution of errors in the population affected by algorithmic driven decisions). Value transparency requires that these normative goals and the reasons for considering them are clearly specified—i.e. the choice of such normative goal is not a mere arbitrary decision by the data scientists. It contributes to the ability of the public to understand and assess the validity of a potential justification for accepting decisions taken by a model pursuing such goal. If the goals and constraints pursued by a model do not reflect values worth pursuing, the decisions following from the model are not justified.

An algorithm pursuing such goals will achieve them to a determined degree, which is expressed by “performance transparency.” The performance can only be assessed by translating the goals in question into measurable quantities. This exercise of translation is not trivial. With reference to the FR example above, the translation of a moral constraint (e.g. anti-discrimination) into a quantifiable performance measure (e.g. equality in the false positive and false negative rate across racial and sex groups) should be given a normative grounding, and not simply be assumed. If the value translation is not declared and no reason to accept it is given, the decisions of the model are not even *prima facie* justified.

Performance transparency is especially important when there are trade-offs between different values simultaneously pursued by a model. Performance metrics provide an important indication of the extent to which every value has been achieved, which is especially important for the overall justification of the system when a value can only be achieved at the expense of another value. For example, fairness can only be pursued at the expense of efficiency (Corbett-Davies et al. 2017; Wong 2019). Performance transparency provides an indication of the degree to which both values, of efficiency and (quantified) fairness have been sacrificed.

Notice that *design publicity* does not require that individuals that are accountable for algorithmic decisions provide fully persuasive and non-corrigible justifications. It is sufficient that they declare what they take to be the relevant elements, exposing themselves to public scrutiny, as the above-mentioned FR case exemplifies. As Pak-Hang Wong observes “the idea of […] algorithmic fairness is […] contestable […] there is a great number of definitions of what […] algorithmic fairness amounts to, and it seems unlikely for researchers […] to settle on *the* definition of fairness any time soon” (Wong 2019). Design publicity is intended to empower the public to debate also such choices, so as to enable their revision. This is compatible with the idea of the perfectibility of the public justification of algorithms over time, which is what we intend to enable through design publicity.

There is still a gap in the justification of individual decisions. As anticipated, the fact that prediction-based decisions will often be wrong can be justified. In the case of machine learning-driven algorithms, individual mistreatment happens because the information necessary to always make perfect predictions does not exist. And even the information required to make a model *more* accurate may be too costly to collect, or cannot be collected in morally permissible ways. It is known that value-driven design that considers privacy and non-discrimination pays a price in terms of predictive accuracy (Hajian et al. 2015) and efficiency (Corbett-Davies et al. 2017). A further reason why errors are unavoidable is that some outcomes result from human free will, for example, success during parole. The same considerations (of cost, privacy, or fairness) justify statistical decisions that rely on incomplete information, even when it is theoretically possible to collect and analyze all the information that matters, in principle, if one is to treat each individual case “as a distinct individual” (Lippert-Rasmussen 2010).

An individual subjected to an unfavorable decision may accept, in principle, that the algorithm is justified as a whole, yet challenge the *necessity* of implementing the model when taking a decision *about him*. The particular individual may argue: “I understand that the algorithm achieves these goals and that it does so in a reasonable way. But why can’t you make an exception for me?”. This would violate consistency. For example, suppose that a software is used to randomize access to scarce life-saving resources in a hospital of a dystopian country. This software translates fairness into a basic mathematical condition, which is equal chances of getting the resource in question. This goal can be achieved by an algorithm whose outcome is completely random. Yet consistency would be violated if, when the case of the head physician’s son is submitted to it, the randomized model is no longer used by the person in charge, who recognizes the head physician’s son, and assigns the resource to him. In this case, the software does not satisfy consistency.

The violation of consistency *for an arbitrary reason* (e.g. the case of the head physician's son) is incompatible with equal respect; on the other hand, if the same exception were made for everyone who had an interest to demand it, the algorithm wouldn’t achieve its design goals, which justify its use. The violation of consistency is incompatible with formal justice, i.e., “the impartial and consistent administration of laws and institutions” [29], applied to the algorithm, considered as a law, or as an institution. This is why—we maintain—algorithms that change their identity as they are used are normatively problematic in high-stake decisions. In such cases, any change due to retraining should be at least publicized, and justified, by pointing out a considerable improvement in performance, which overrides consistency concerns.

When the design of an algorithm is justified, then, if the algorithm is also used consistently, we obtain a *procedural justification* of all the decisions that follow from it. To explain this kind of justification, we draw from Rawls’s idea of the justification of individual shares of the goods produced by cooperation (Rawls 1999). Rawls rejects the idea of allocative justice, namely, he rejects describing justice as a property of the end-state of process of the distribution of goods, a property independent from how that distribution came about. For example, an end-state distribution is just, according to a resource egalitarian account, if and only if resources are equally distributed, according to a meritocratic account, if and only if resources are proportional to each person’s contribution to society, and according to a utilitarian one, if and only if the distribution maximizes utility. As Nozick (1974) observes, these allocative end-states are undermined by processes, like markets, that are not fully deterministic, because they are perturbed by human free decisions. In Nozick’s slogan, liberty upsets patterns. Importantly, this applies to many cases of algorithmic decision-making, where the outcomes that justify the decision are future events that depend on the free will of an individual. According to non-compatibilist libertarianism, for example, believing that an inmate success on parole could be predicted with perfect precision is tantamount to denying that the inmate has free will.

This gives us a moral reason to consider Rawls’s procedural alternative to end-state conceptions. In this case, distributive shares are just if they result from just institutions. But unlike Nozick, Rawls relates the justification of institutions to the outcomes they tend to bring about, their general statistical tendencies, considered from a suitably general perspective. As in Hume it is the “general scheme or system of action, which is advantageous” (Hume et al. 2000), not every single decision is considered individually. The outcomes which justify the institutions are characterized by Rawlsian principles of justice. Rawls (Rawls 1999), for examples, requires economic institutions as a whole (including taxation) to maximize the expectations of the worst off groups in society. If institutions are justified, and if they are consistently and impartially applied, then the outcomes of free human decisions constrained by institutions are just, whatever they are.

For algorithmic decision-making, the principles of justice correspond to its design goals. The design goals of the algorithm are *that which justifies an algorithm which amounts to specific rules* (including inscrutably complex ones). To assess if inscrutable algorithms satisfy their “principles of justice” we consider their *performance*. If they do, the consistent and impartial application of the algorithm to individual cases corresponds to the consistent and impartial administration of just institutions. Summing up in one word: we are bound *by procedural justice* to accept as just *only consistent decisions* that result from the application of an algorithm that is justified by design.

Notice that, in the institutional case, the fact that institutions are administered consistently and impartially is a *public* fact. This publicity is achieved thanks to special procedures. E.g. the consistent fulfillment of the legal obligations emerging from civil law can be tested by going to court. In the algorithmic case, the consistent application of *inscrutably complex rules* appears to lack transparency. The solution to this is to provide a technical solution that delivers a proof that the rules are followed—that is, consistency—even when the rules themselves are not transparent to anyone because the algorithm is a black box; it appears that this is indeed technically feasible (Kroll et al. 2017).

## Conclusion

In this paper, we discuss what it means to achieve *transparency* for machine learning algorithms, i.e. the provision of explanations to see through, analyze, and assess artifacts trained on data via machine learning methods and generating predictions to assist or automate decision-making. We propose a form of transparency that consists in publicizing the *design* of an artifact (including *value*, *translation* and *performance*) as well as its *consistent* application. We maintain that this kind of transparency provides (1) an *explanation* of the artifact, namely, an explanation “by design”; (2) an *intentional* explanation of its deployment; (3) a justification of its use; (4) when used consistently, a *procedural* justification of the individual decisions it takes.

The proposed approach to algorithmic transparency deviates from the existing body of literature on explainable artificial intelligence (xAI), where the concept of transparency focuses on the explanation of the inner workings of algorithms or the interpretability of their individual outcomes (Lipton 2018; Ribeiro et al. 2016). We do not claim here that transparency as design publicity achieves the goals that these approaches are said to achieve. Rather, we stress that transparency as design publicity achieves a distinct goal, namely, providing the public with the essential elements that are needed in order to assess the justification (and, when consistency is satisfied, procedural justice) of the decisions that follow from its deployment.

## Footnotes

1 This is a much weaker, open ended, and vague account of public justification than the one offered by Binns (2018, p. 554) according to which a public justification must be grounded in public reason, implying that it “﻿must be able to account for its system in normative and epistemic terms which all reasonable individuals in society could accept” For society, the account of what counts as *public* argument (i.e. in our view, one involving reasons relevant from an impartial standpoint) is meant to be as revisable and open-ended as every other assumption on which the justification of algorithms rests.

2 Additionally, counterfactual explanations may provide users with scenarios, which cannot be realized in practice, as they violate, for example, the causal constraints between model features used to generate the explanation (“lack of ontological stability”). In a big-data context, i.e. in presence of hundreds or thousands of variables and synthetic data points, hard-coding causal constraints in the synthetic data generating algorithm that reflect a priori criteria of plausibility or possibility is an unviable strategy, due to the time needed for considering and implementing all possible scenarios.

3 Moreover, like other post-hoc explanations, counterfactual explanations provide only local explanations of selected machine learning outcomes; moreover, the choice of the features to highlight may reflect subjective and potentially opaque preferences of the person in charge for providing counterfactual explanations to those demanding them (“selection bias”), and are sensitive to perturbations of input data (“lack of robustness” (Hancox-Li 2020)).

4 Where clearly, “each type of feature-highlighting explanation may define “matter” differently” (Barocas et al. 2020, p. 81).

5 By public acceptability we do not mean public in the sense of Rawlsian public reason (Binns 2018; Rawls 1996), which involves standards of justification which can be shared by individuals with different conceptions of the good sharing a commitment to core liberal and democratic values and principles. We assume that different standards of justification will be employed in different contexts and by different publics.

6 The final cause described by Aristotle can be used to explain the behavior of entities with no psychological states (desires, beliefs, conscious purposes, etc.) such as algorithmic systems, as Aristotle applies the teleological model of explanation to natural processes, which have no psychological states ((Broadie 1987), (Gotthelf 1976)).

7 What counts as a satisfactory in a given context may vary from context to context, also depending on the stakes of public justification, discussed in Sect. 4.

8 To churn or to lapse is the activity of moving out a given group. In business, it refers to the activity of customers to move out of portfolios. Predictive models of customer churns are important to organizations to predict the volumes of portfolios in (future) timeframes and to assess their (future) profitability.

9 Training and test data are often the result of a random split of an original set of data used for modelling purposes. This implies that the object resulting from training and the outcomes of which are the object of the explainability analysis is in reality a *pair* consisting of the model and a random seed, which is the integer value chosen by the analyst that governs the *randomness* in the routines leading to the training of the model itself.

10 This three-level approach has a similar structure to that of the incremental model designed by Castelluccia and Le Mètayer Inria (2020). This model provides a methodology for assessing the impact of face recognition (FR) systems and is constituted by four levels of analysis: the goal of the system, the means to achieve it, the suitability of the use of FR systems to achieve the means, and the suitability of a specific technology to achieve the means. However, the objective and scope of our approach are different: while Castelluccia and Le Mètayer Inria propose a model for evaluating all the potential impacts of FR systems (and more in general AI) on society, we provide a theoretical tool for evaluating the transparency of ML models and whether their use is justified within a society. Furthermore, a comparable three-level distinction was employed in safety testing by DeepMind (Ortega et al., 2018). The DeepMind approach distinguishes three concepts of specification, corresponding to what we call “value”, “translation”, and “performance”, which are respectively the ideal specification (the general description of an ideal AI system that is fully aligned to the desires of the human operator), the design specification (the specification that is actually used to build the AI system), and the revealed specification (the specification that best describes the system’s behavior). However, unlike our model, DeepMind applies these three levels specifically to the security tests that an AI company should conduct as an internal practice, and it does not intend these levels to be used to describe the system for the sake of transparency.

11 The alternative being a performance measure assigning a different weight to the avoidance of type-I and type-II errors (Kraemer et al 2011; Corbett-Davies et al 2017).

12 The multi-faceted nature of transparency of algorithms is discussed in (Pégny & Ibnouhsein, 2018), where the authors describe the distinction between its epistemic (e.g. intelligibility and explicability) and normative (e.g. loyalty and fairness) *desiderata*.
